# Biopreservation of Chocolate Mousse with *Lactobacillus* *helveticus* 2/20: Microbial Challenge Test

**DOI:** 10.3390/molecules27175631

**Published:** 2022-08-31

**Authors:** Bogdan Goranov, Desislava Teneva, Rositsa Denkova-Kostova, Vesela Shopska, Nadia Oulahal, Zapryana Denkova, Georgi Kostov, Pascal Degraeve, Rafael Pagan

**Affiliations:** 1 Department “Microbiology”, University of Food Technologies—Plovdiv, 26 Maritza Boulevard, 4002 Plovdiv, Bulgaria; 2 Laboratory of Biologically Active Substances, Institute of Organic Chemistry with Centre of Phytochemistry, Bulgarian Academy of Sciences, 139 Ruski Boulevard, 4000 Plovdiv, Bulgaria; 3 Department “Technology Biochemistry and Molecular Biology”, University of Food Technologies—Plovdiv, 26 Maritza Boulevard, 4002 Plovdiv, Bulgaria; 4 Department “Technology of Wine and Beer”, University of Food Technologies—Plovdiv, 26 Maritza Boulevard, 4002 Plovdiv, Bulgaria; 5 Bioingénierie et Dynamique Microbienne aux Interfaces Alimentaires Research Unit, IUT Lyon 1, Technopole Alimentec, Université Claude Bernard Lyon 1, ISARA Lyon, 155 rue Henri de Boissieu, F-01000 Bourg en Bresse, France; 6 Departamento de Producción Animal y Ciencia de los Alimentos, Facultad de Veterinaria, Instituto Agroalimentario de Aragón-IA2, Universidad de Zaragoza-CITA, Calle Miguel Servet, 177, 50013 Zaragoza, Spain

**Keywords:** biopreservation, chocolate mousse, microbial challenge test, probiotics

## Abstract

Probiotic bacteria are used for food biopreservation because their metabolic products might contribute to ensuring food microbiological safety and/or increase its shelf life without the addition of chemical preservatives. Moreover, biopreserved foods are excellent vehicles for the delivery of probiotic bacteria. The aim of the study was to investigate the potential of chocolate mousse food matrix for the delivery of the probiotic strain *Lactobacillus* *helveticus* 2/20 (*Lb. helveticus* 2/20) and to investigate its capacity to inhibit the growth of two foodborne pathogenic bacteria (*Staphylococcus* *aureus* and *Escherichia* *coli*). Therefore, the populations of free or encapsulated in calcium alginate *Lb. helveticus* 2/20 cells and/or of each pathogen (used to voluntarily contaminate each sample) were monitored both in complex nutrient medium (MRS broth) and in chocolate mousse under refrigeration conditions and at room temperature. *Lb. helveticus* 2/20 alone in free or encapsulated state effectively inhibited the growth of *Escherichia* *coli* ATCC 25922 and *Staphylococcus* *aureus* ATCC 25923 in chocolate mousse when stored at 20 ± 2 °C. Practically no viable unwanted bacteria were identified on the 7th day from the beginning of the process. High viable *Lb. helveticus* 2/20 cell populations were maintained during storage under refrigerated conditions (4 ± 2 °C) and at room temperature. Chocolate mousse is thus a promising food matrix to deliver probiotic *Lb. helveticus* 2/20 cells, which could also protect it from contamination by unwanted bacteria.

## 1. Introduction

Biopreservation is a method of food quality preservation and shelf-life extension by introducing antimicrobial substances of plant origin, animal origin or microorganisms, producing metabolites with high antimicrobial activity [1,2,3]. Thus, biopreservation is a natural way to protect foods from spoilage and harmful contamination. The food industry is presently looking for means of producing safe food products with an extended shelf life in order to meet the consumer demands for natural, low salt, low sugar foods and with reduced use of chemical preservatives. One approach to solve the problem is fermentation. Fermented food products have a longer shelf life and are less prone to spoilage than fresh food products of the same matrix [4]. For a variety of food products, fermentation is not a suitable biopreservation strategy due to a few reasons. First of all, not all food matrices can undergo fermentation. Secondly, sometimes the aim behind preservation is to not change any organoleptic characteristic of the final product. Thus, sometimes microorganisms are added to foods not to perform fermentation but to exert protective effect on the safety of the food products.

Probiotic bacteria (lactobacilli and/or bifidobacteria) hold a promising potential to be used as part of successful strategies for food biopreservation. The added probiotic starter cultures participate in the formation of food taste and aroma, but also serve as food biopreservatives, to increase their shelf life. In addition, incorporated probiotic cultures turn food into functional food; i.e., probiotics in food ensure its biopreservation and increase its functionality. This in turn is related to their beneficial effect on the human body [5,6,7]. Lactic acid bacteria (LAB) and other food microorganisms produce a wide range of metabolites that can inhibit growth of spoilage and pathogenic bacteria and act as multiple hurdles. The LAB antimicrobial activity has been attributed to the production of metabolites, such as organic acids (lactic and acetic acid), hydrogen peroxide, ethanol, diacetyl, acetaldehyde, other low molecular mass compounds with antimicrobial activity and bacteriocins [8]. The production of weak organic acids, such as acetic and lactic acids, inhibits microbial growth through multiple actions, including membrane disruption, inhibition of metabolic reactions, disturbance of pH homeostasis, and accumulation of toxic anions in the cell [9,10].

As previously stated, to answer the strong societal demand for minimally processed and preservative-free foods, biopreservation has been receiving growing interest as a way to improve food quality and safety. In past years, many strains from various microbial species with antimicrobial properties have been identified. They have been isolated from various sources, such as fruits, vegetables, cereals, milk, meat, and other food-related products as well as human origin [11,12,13]. Screening steps are required to find efficient microorganisms with good antimicrobial activity levels and the spectrum of the inhibited pathogen targets greatly varies depending on the considered species, and from strain to strain within a species [14].

Most in vitro screening experiments of LAB antimicrobial activities have been performed using synthetic media, such as the de Man, Rogosa, and Sharpe (MRS) agar medium [14]. The composition of MRS can have a strong impact on the expression of the antimicrobial activity of LAB because it contains acetate, which may reinforce lactic acid bacteria antimicrobial activity and artificially inflate the number of active isolates. Other important points are the nature and concentration of sugars and the buffering capacity of the medium, which are highly expected to influence the amount of organic acids and the final pH of the medium, thus modifying the antimicrobial activity. Thus, it is important to take into account the conditions prevailing in situ to develop relevant screening approaches [14].

It is extremely important for in situ evaluation of the antimicrobial activity of the microorganisms or their metabolites, selected as a result of in vitro screening, to be conducted in the actual food products, because many studies report discrepancies between results observed in vitro and in situ [14,15].

LAB have been successfully applied either as living cells or their metabolic products (mainly bacteriocins and bacteriocin-like substances) as part of successful strategies for biopreservation of different food products such as meat products [16,17], seafood [18], vegetables [19], bread and bakery products [20], chocolate [21], etc. Every food matrix presents a number of challenges in the development of successful biopreservation strategies. Thus, every biopreservation strategy has to be custom-made for the specific food matrix. One successful biopreservation approach cannot be directly transferred to another food matrix, hoping to be as successful at biopreservation as in the original product. For example, although the use of LAB and their bacteriocins in real meat products at lab scale generally leads to a lower antibacterial activity due to the complexity of the composition and structure of these foods, promising results for a wide range of meat products have been obtained [16,17]. The application of symbiotic sourdough starters for different bread types and bakery products results in the production of food products with more abundant aroma compounds and higher nutritional values compared to foods fermented with yeasts alone. Moreover, bread and bakery products obtained with the use of starter sourdough demonstrate longer shelf life due to the production of various LAB metabolites with antimicrobial activity that inhibit spoilage microorganisms [20]. Different chocolate types have proven to be an adequate vehicle for the delivery of different probiotic strains (*Lactobacillus* sp. and *Bifidobacterium* sp.). The addition of probiotics to chocolate did not affect the physico-chemical properties and sensory acceptability of the final products, thus demonstrating that chocolate is an excellent food matrix to be used as a vehicle for the delivery of beneficial probiotic bacteria. Furthermore, micro-encapsulated probiotics demonstrated higher bacterial viability than probiotics added as freeze-dried powder, clearly suggesting that it would be relevant to perform microencapsulation prior to LAB addition to chocolate formulations. In addition to the nutraceutical properties of the added probiotic strains, they also serve as successful biopreservation agents of food products, thus actually exerting a dual positive effect [21].

Aerated dairy desserts have shown a great market potential as a function of consumer behavior, i.e., interest in lighter and healthier relish products [22,23]. The development of new technologies facilitating the supplementation of confectionery products with probiotic LAB with biopreservation potential can lead to the development of novel functional confectionery products enriched with health-promoting ingredients and with extended shelf life. Since confectionery products, including chocolate mousse, are consumed not only by adults but also by children and teenagers, their supplementation with live probiotic LAB is advisable, as they can serve as excellent probiotic delivery vehicles for all age groups [23].

“Mousse” (from the French word meaning “foam”) is a light dessert with an airy structure. It originates from France, and the information about the first mousse dates back to the 18th century. The most popular mousse is chocolate mousse, followed by orange mousse, lemon mousse and strawberry mousse. The process of preparation of the chocolate mousse begins with mixing the chocolate with water or milk and melting it in a water bath with constant stirring until a smooth suspension is obtained. Raw egg yolks are then added, and with constant stirring, the mixture is added to the whipped egg whites and/or cream and allowed to cool. The mousse preparation requires time and skill so as not to turn the final product into an unattractive, sticky mass. The microorganisms in these products come from their ingredients, equipment and air [23].

*Lb. helveticus* strains with proven probiotic properties are excellent candidates for application in biopreservation strategies for different food products. The application of the probiotic *Lb. helveticus* strain in the present research aimed at suppressing and controlling the growth of the test pathogens as a result of its vital activity and metabolites produced and secreted in the environment. Several authors encapsulated LAB in calcium alginate beads before their addition to foods [24,25]. Entrapment of LAB cells in calcium alginate beads can preserve their viability and their activity [26]. Therefore, in the present study, the same amount of viable probiotic *Lb. helveticus* 2/20 cells were used to inoculate chocolate mousse or MRS broth either directly, or following their encapsulation in the form of calcium alginate particles.

The aim of the study was to investigate the potential of chocolate mousse food matrix for the delivery of the probiotic strain *Lactobacillus helveticus* 2/20 (*Lb. helveticus* 2/20) and to investigate its capacity to inhibit the growth of two foodborne pathogenic bacteria *Staphylococcus aureus* ATCC 25923 and *Escherichia coli* ATCC 25922 in MRS broth nutrient medium and in chocolate mousse food matrix.

## 2. Materials and Methods

### 2.1. Microorganisms

In order to investigate the potential of the chocolate mousse food matrix for the delivery of the probiotic strain *Lactobacillus helveticus* 2/20 (*Lb. helveticus* 2/20) and to investigate its capacity to inhibit the growth of two foodborne pathogenic bacteria *Staphylococcus aureus* ATCC 25923 and *Escherichia coli* ATCC 25922 in MRS broth nutrient medium and in chocolate mousse food matrix, the following two microorganism group were used: (1) *Lb. helveticus* 2/20 probiotic strain that was isolated from the rose blossom of *Rosa damascena* Mill L. The strain had been identified using biochemical (API 50 CHL) and molecular-genetic (sequencing of the 16S rDNA) methods (unpublished data). It was cultivated in MRS broth. (2) *Escherichia coli* ATCC 25922 (*E. coli* ATCC 25922; a Gram-negative bacterium), and *Staphylococcus aureus* ATCC 25923 (*S. aureus* ATCC 25923; a Gram-positive bacterium) were used as test microorganisms to investigate the capacity of *Lb. helveticus* 2/20 strain to control these foodborne pathogenic bacteria. *E. coli* ATCC 25922 and *S. aureus* ATCC 25923 strains were cultured at 37 °C in LBG broth medium.

### 2.2. Nutrient Media

#### 2.2.1. Normal Saline Solution (0.9% (*w*/*v*) NaCl)

Saline solution was used for the preparation of ten-fold serial dilutions of the different samples and for the biomass washing in the encapsulation procedure.

#### 2.2.2. MRS Broth

Composition (g/L): peptone proteose, 10.0; yeast extract, 4.0; meat extract, 8.0; glucose, 20.0; K_2_HPO_4_, 2.0; sodium acetate, 3H_2_O, 5.0; triammonium citrate, 2.0; MgSO_4_, 7H_2_O, 0.2; MnSO_4_, H_2_O, 0.05; Tween® 80, 1 mL. The final pH was adjusted to 6.5 using 1M NaOH. The medium was used in the in vitro determination of the antimicrobial activity of *Lb. helveticus* 2/20 against pathogenic microorganisms and as a medium for culturing *Lb. helveticus* 2/20.

#### 2.2.3. LAPTg10 Agar

Composition (g/L): peptone, 15, yeast extract, 10; tryptone, 10, glucose, 10. The pH is adjusted to 6.6–6.8 using 1M NaOH and Tween® 80, 1 mL, was added. The medium was used for the determination of the number of viable lactobacilli cells.

#### 2.2.4. LBG Broth

Composition (g/L): tryptone, 10.0; yeast extract, 5.0; NaCl, 10.0; glucose, 10.0. The final pH was adjusted to 7.5 using 1M NaOH. The medium was used for culturing *E. coli* ATCC 25922 and *S. aureus* ATCC 25923.

#### 2.2.5. LBG Agar

LBG-agar medium: Composition (g/L): tryptone, 10.0; yeast extract, 5.0; NaCl, 10.0; glucose, 10.0; agar, 20.0. The final pH was adjusted to 7.5 using 1M NaOH. The medium was used for the determination of the number of viable pathogen (*E. coli* or *S. aureus*) cells.

All media were autoclaved for 20 min, at 121 °C. All nutrient media were of analytical grade and were from Merck^®^.

### 2.3. Experimental Procedures and Methods of Analysis

#### 2.3.1. Determination of Biochemical Profile of Lactobacillus 2/20

The API 50 CHL system (BioMerieux® SA, Marcy-l’Étoile, France) was used to identify Lactobacillus strain 2/20 based on the consumption of 49 carbon sources. The kit was used according to the instructions of the manufacturer.

#### 2.3.2. Molecular-Genetic Identification of Lactobacillus 2/20: Sequencing of the 16S rRNA Gene of Lactobacillus 2/20

The sequencing of the 16S rRNA gene was performed by the Sanger method at Macrogen Europe Laboratory, The Netherlands. The forward and reverse partial sequences obtained from the 16S rRNA gene sequencing with the universal primers 27F and 1492R were assembled using *CLC Sequence Viewer* software. The whole 16S rRNA gene sequence was compared with the sequences available in the GenBank online database using online BLASTn software, and the species affiliation of *Lactobacillus* 2/20 was determined with the corresponding similarity percentage between the sequence of *Lactobacillus* 2/20 and the reference strain from the online database.

#### 2.3.3. Biopreservation Assay

In order to investigate the capacity of added *Lb. helveticus* 2/20 cells to control the growth of two pathogens, chocolate mousse and MRS broth were artificially contaminated either with a *S. aureus* strain, or with an *E. coli* strain. Control experiments with chocolate mousse or MRS broth only inoculated with *Lb. helveticus* 2/20 cells or each pathogen strain allowed us to estimate the interaction between *Lb. helveticus* 2/20 and each of the pathogens in each system and during storage at 4 ± 2 °C to mimic refrigeration or at 20 ± 2 °C to mimic a break of cold chain.

##### Encapsulation of Lactic Acid Bacteria in Calcium Alginate Particles [24]

Fresh 24 h culture of *Lb. helveticus* 2/20 was centrifuged at 5000× *g* for 15 min, at 4 °C. The culture medium was discarded, and the biomass was washed once with saline solution. The biomass was then resuspended to the initial volume with saline solution and was added to 20 mL of 4% (*w*/*v*) sodium alginate. Then, 0.2% (*v*/*v*) Tween® 80 and 20 mL of 4% (*w*/*v*) sodium alginate with LAB were added to 100 mL of sunflower oil, and then the mixture was mixed with a magnetic stirrer for 10–15 min. An amount of 100–200 mL of 2% (*w*/*v*) CaCl_2_ was added to the mixture in portions. The emulsion was stirred for 30 min to gel the alginate, then centrifuged at 5000× *g* for 15 min, at 4 °C. The pellet was washed once with saline solution. The resulting preparation was mixed with 1–2 mL of saline solution and was added to the chocolate mousse or MRS broth.

##### Preparation of Chocolate Mousse

Chocolate mousse was prepared from 2 main ingredients: dark chocolate with a cocoa content of 52% and whipped cream. Then, 400 g of chocolate was melted at 65 °C, stirring periodically. The melted chocolate was left to cool to 45 °C, making sure that it did not start to harden. Meanwhile, 600 mL of pasteurized cream (22% (*w*/*v*) fat) was whipped using a mixer. The slightly cooled chocolate was added to the whipped cream in small portions, stirring constantly to achieve the desired consistency. The chocolate mousse thus obtained was divided into 9 portions of 50 g.

##### Investigation of the Capacity of *Lb. helveticus* 2/20 to Control Unwanted Bacteria

A. Investigation in MRS Broth

Three probes with a 100 mL volume were prepared: (1) *Lb helveticus* 2/20 control, containing 95 mL of MRS broth and 5 mL of a *Lb. helveticus* 2/20 saline solution biomass suspension (with initial microbial load of 2.8 × 10^11^ cfu/mL, obtained from an overnight culture of this strain); (2) pathogen control, containing 95 mL of MRS broth and 5 mL of the saline solution biomass suspension of the respective pathogen; (3) mixture, containing 90 mL of MRS broth, 5 mL of the *Lb. helveticus* 2/20 saline solution biomass suspension (with initial microbial load of 2.8 × 10^11^ cfu/mL, obtained from an overnight culture of this strain) and 5 mL of the saline solution biomass suspension of the respective pathogen (prepared by suspending biomass, grown on solid nutrient medium (LBG agar)). Each probe was divided into two aliquots so that two identical sets of probes were formed. The first set of probes was stored under static conditions, at room temperature (20 ± 2 °C), for 7 days, taking samples at the 0th, 3rd, 5th and 7th day, monitoring the changes in the concentration of viable cells of *Lb. helveticus* 2/20 and the respective pathogen as well as in the pH. The second set of probes was stored under refrigeration conditions (4 ± 2 °C) for 21 days, taking samples at the 0th, 7th, 14th and 21st day, monitoring the concentration of viable cells of *Lb. helveticus* 2/20 and the respective pathogen. The concentration of viable cells of both *Lb. helveticus* 2/20 and the respective pathogenic bacteria and the titratable acidity were monitored. The number of viable cells was determined by preparing appropriate ten-fold dilutions and spread plating on LAPTg10 agar (for the enumeration of *Lb. helveticus* 2/20) and on LBG agar (for the enumeration of the respective unwanted bacteria). The titratable acidity was determined by titration method according to standard protocol [27].

B. Determination of the Antimicrobial Activity Against Pathogenic Microorganisms by Co-cultivation in Chocolate Mousse (Microbial Challenge Test)

Two identical sets of 9 chocolate mousse samples were prepared using 0.25 mL of the biomass suspensions of *Lb. helveticus* 2/20, *S. aureus* ATCC 25923 and *E. coli* ATCC 25922 according to the samples list in Table 1. The initial bacterial load of the *Lb. helveticus* 2/20 suspension was 3.5 × 10^10^ cfu/mL, while the initial bacterial load of the suspension of each pathogen was 4.7 × 10^10^ cfu/mL. The first set of samples was stored under static conditions, at room temperature (20 ± 2 °C), for 7 days, taking samples at the 0th, 3rd, 5th and 7th day, monitoring the concentration of viable cells of *Lb. helveticus* 2/20 and of the respective pathogen as well as the pH. The second set of samples was stored under refrigeration conditions (4 ± 2 °C) for 21 days, taking samples at the 0th, 7th, 14th and 21st day, monitoring the concentration of viable cells of *Lb. helveticus* 2/20 and of the respective pathogen as well as the pH. The number of viable cells was determined by preparing appropriate ten-fold dilutions and spread plating on LAPTg10 agar (for the enumeration of *Lb. helveticus* 2/20) and on LBG agar (for the enumeration of the respective pathogen). The pH was determined potentiometrically.

The preparation of chocolate mousse variants with either free or encapsulated cells aimed at giving insight on whether the inclusion of encapsulated cells would lead to retaining higher concentrations of viable probiotic lactobacilli cells during the studied storage periods at the respective temperature. The very aim of the biopreservation strategy under development was both to ensure the safety of the chocolate mousse and to turn the chocolate mousse food matrix into a vehicle for the delivery of high concentrations of probiotic lactobacilli cells.

Determination of the Number of Viable Microorganism Cells, the Growth Rate and the Death Rate of Microorganisms and the Activation Energy 

Appropriate ten-fold dilutions of each sample in saline solution were prepared. They were used for spread plating on LBG agar for determining the viable counts of the pathogens and on LAPTg10 agar for determining the concentration of viable lactobacilli cells. The inoculated plates were incubated for 3 days, at 37 ± 1 °C, until the appearance of countable single colonies (between 1 and 30).

In order to reduce the influence of dimensionality in the processing of the results obtained, due to the different biological nature of the microorganisms used, the results obtained were converted into dimensionless form as follows [28]:

(1) $$DB=\frac{X_{i}}{X_{0}}$$

where: *DB* is the biomass in dimensionless form; *X_i_* is the current concentration of the biomass of the respective microorganism, cfu/mL; and *X_0_* is the initial biomass concentration of the respective microorganism, cfu/mL.

The specific growth rate of microorganisms (lactobacilli and pathogenic microorganisms) was determined according to Equation (2) [29]:

(2) $$\frac{dX}{d\tau }=\mu X-\beta X^{2}$$

where: *X* is the biomass concentration, cfu/mL; *μ* is the specific growth rate, h^−1^; and *β* is the internal population competition coefficient, cfu/(mL.h).

The specific death rate of microorganisms (lactobacilli and pathogenic microorganisms) was determined according to Equation (3) [29]:

(3) $$\frac{dX}{d\tau }=-kX$$

where: *X* is the biomass concentration, cfu/mL; and *k* is the specific death rate of the microorganisms, h^−1^.

Activation energy and pre-exponential multiplier Z in the Arrhenius equation were also determined according to Equations (4) and (5) [30]:

(4) $$E_{a}=\frac{\ln\frac{{µ}_{max2}}{{µ}_{max1}}\cdot RT_{1}T_{2}}{\left(T_{2}-T_{1}\right)}$$

where: *Ea* is the activation energy of growth, kJ/mol; *R* is the universal gas constant (*R* = 8.314 J mol^−^^1^ K^−^^1^); *µ_max1_* and *µ_max2_* are the maximum specific growth rate (h^−1^) at 4 ± 2 °C and 20 ± 2 °C, respectively; and *T_1_* and *T_2_* are the storage temperatures (K).


(5) $${µ}_{max}={Ze}^{-E_{a}/\mathrm{RT}}$$

### 2.4. Processing of the Results

Data from triplicate experiments were processed using MS Office Excel 2013 software, using statistical functions to determine the mean values, standard deviation and maximum estimation error at significance levels of *p* < 0.05.

## 3. Results and Discussion

### 3.1. Identification of Lactobacillus 2/20

Based on the studies conducted with the API 50 CHL system (Table 2), *Lactobacillus* 2/20 was identified as a representative of the species *Lactobacillus helveticus* with the corresponding percentage of confidence being 99.9%.

**Figure 1:**
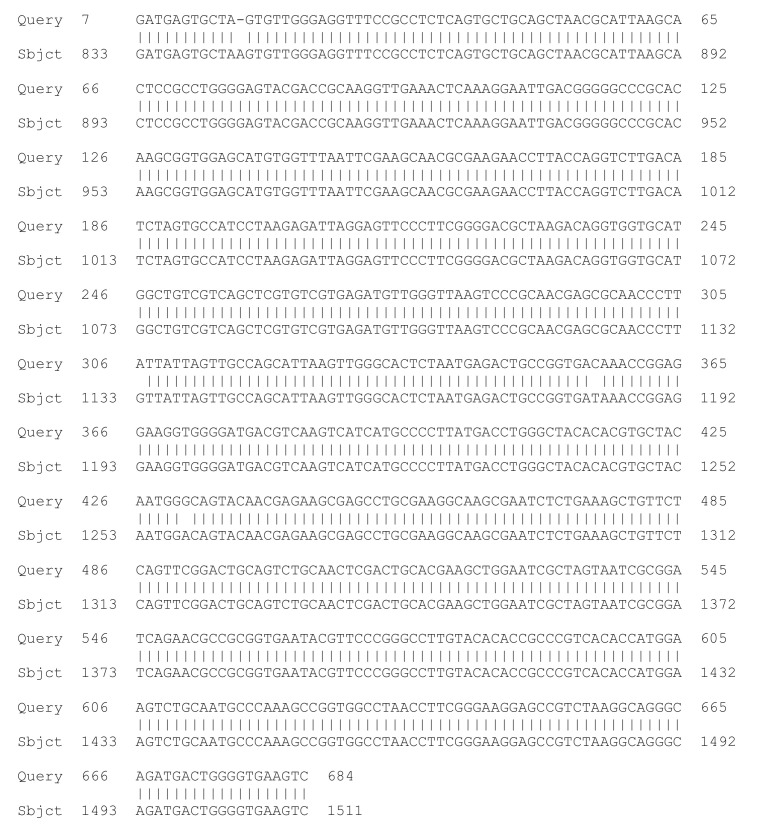
Comparison between the partial nucleotide sequence of the 16S rDNA of *Lactobacillus* 2/20 and the partial nucleotide sequence of the 16S rDNA of *Lactobacillus helvetucus* DSM 20075.

A molecular genetic method for genotyping—sequencing of the 16S rRNA gene was used to confirm the species identification of *Lactobacillus* 2/20. The results of the 16S rDNA sequencing analysis referred *Lactobacillus* 2/20 to the species *Lactobacillus helveticus* with 99% similarity between the 16S rDNA sequence of *Lactobacillus* 2/20 and the partial 16S rDNA sequence of *Lactobacillus helveticus* DSM 20075 (Figure 1). 

The conducted biochemical and molecular-genetic identification of *Lactobacillus* 2/20 referred the studied strain to the species *Lactobacillus helveticus*.

### 3.2. Biopreservation of Chocolate Mousse: Determination of the Survival of Lactobacilli and Pathogenic Bacteria during Storage at Temperatures of 4 ± 2 °C and 20 ± 2 °C

In order to estimate the capacity of *Lb. helveticus* 2/20 cells to protect chocolate mousse from contamination by either *S. aureus* ATCC 25923 or *E. coli* ATCC 25922, MRS broth and chocolate mousse inoculated with either free or encapsulated *Lb. helveticus* 2/20 cells were artificially contaminated with either *S. aureus* ATCC 25923 or *E. coli* ATCC 25922 cells.

The results of the determination of the kinetic characteristics of growth and death of biomass are presented in Table S1 (Supplementary Materials).

- Biopreservation of Chocolate Mousse with Free or Encapsulated *Lb. helveticus* 2/20 Cells

Monitoring of *Lb. helveticus* 2/20 and *E. coli* ATCC 25922 populations and of the total titratable acidity of chocolate mousse and MRS broth during storage at 4 ± 2 °C and 20 ± 2 °C.

**Figure 2:**
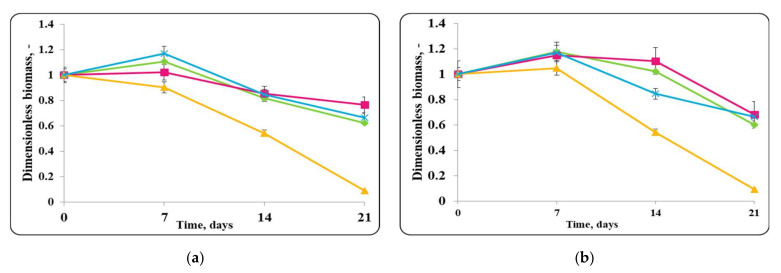
(**a**) free lactobacilli cells; (**b**) encapsulated lactobacilli cells. Changes in the dimensionless biomass of *Lb. helveticus* 2/20 and *E. coli* ATCC 25922 in chocolate mousse at 4 ± 2 °C. Legend: **Green** ◆: *Lb. helveticus* 2/20 population following its inoculation; **Pink** **■**: *Lb. helveticus* 2/20 population following its co-inoculation with *E. coli* ATCC 25922; **Blue** **×**: *E. coli* ATCC 25922 population following its direct inoculation; **Yellow** **▲**: *E. coli* ATCC 25922 population following its co-inoculation with *Lb. helveticus* 2/20.

The changes in the dimensionless biomass in the chocolate mousse with free or encapsulated *Lactobacillus helveticus* 2/20 cells during storage at 4 ± 2 °C were similar to those in MRS broth at the same temperature (Figure 2). In the case of the chocolate mousse, a slight increase in the biomass during the first 7 days of refrigerated storage was observed. Then, there was a slight decrease in the cell number or retained growth.The initial *Lb. helveticus* 2/20 concentration in the chocolate mousse samples was 10^8^ cfu/g. Interestingly, even after 21 days storage at 4 ± 2 °C, chocolate mousse always had a *Lb. helveticus* 2/20 population exceeding 10^6^ cfu/g, which is the minimal population of probiotic bacteria in fermented milk according to French regulation [31].

**Figure 3:**
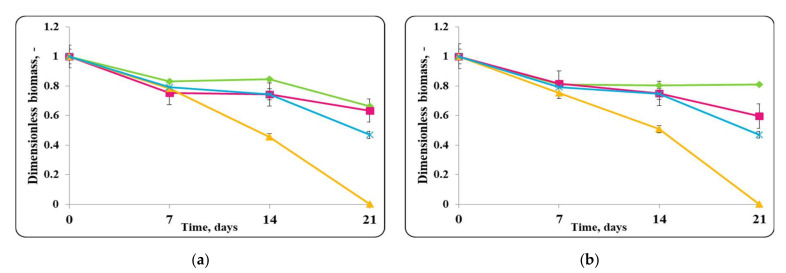
(**a**) free lactobacilli cells; (**b**) encapsulated lactobacilli cells. Changes in the dimensionless biomass of *Lb. helveticus* 2/20 and *E. coli* ATCC 25922 in MRS broth at 4 ± 2 °C. Legend: **Green** ◆: *Lb. helveticus* 2/20 population following its inoculation; **Pink** **■**: *Lb. helveticus* 2/20 population following its co-inoculation with *E. coli* ATCC 25922; **Blue** **×**: *E. coli* ATCC 25922 population following its direct inoculation; **Yellow** **▲**: *E. coli* ATCC 25922 population following its co-inoculation with *Lb. helveticus* 2/20.

The data presented in the figures show that MRS broth did not support the growth of both free and encapsulated lactobacilli and *E. coli* ATCC 25922 under refrigerated storage conditions (4 ± 2 °C), likely due to the low temperature (Figure 3). A gradual reduction in the number of LAB (inoculated as free or encapsulated cells) with comparable death rate constants—0.012 h^−1^ for lactobacilli in the samples with free cells and 0.009 h^−1^ for lactobacilli in the samples with encapsulated cells—was observed from the very beginning of the storage period (Figure 3). A similar trend was observed in the control sample with *E. coli* ATCC 25922, which was expressed in the gradual reduction in the pathogen cells from the very beginning of the storage period with a death rate constant of 0.025 h^−1^, and the concentration of viable *E. coli* ATCC 25922 cells decreased to about 10^7^ cfu/mL at the end of the storage period, which significantly exceeded the minimum infectious concentration of active cells of the pathogen (10^5^ cfu/mL). However, a concentration of active lactobacilli cells of about 10^7^ cfu/mL for the samples inoculated with free cells and about 10^8^ cfu/mL for the ones inoculated with encapsulated cells was maintained in the MRS broth medium up to the end of the storage period. The lower death rate and the higher concentration of active lactobacilli cells (by one logarithmic unit) in the samples inoculated with encapsulated cells was due to the protective effect of the immobilization matrix on LAB. A decrease in the concentration of active lactobacilli cells from the beginning of the process with the same death rate of 0.017 h^−1^ in the mixed populations of free or encapsulated lactobacilli and *E. coli* ATCC 25922 was observed, and the final concentration of active lactobacilli was about 10^7^ cfu/mL. However, for *E. coli* ATCC 25922 in the mixed populations with free or encapsulated lactobacilli, complete reduction in the active cells of the pathogen was observed with comparable death rates, 0.062 h^−1^ for the mixture with free lactobacilli cells and 0.067 h^−1^ for the mixture with encapsulated lactobacilli cells (Figure 3). Other authors examined the antimicrobial activity of a number of LAB against *E.coli* during storage at different temperatures including refrigerated storage. The present observations are consistent with Aguilar et al. (2010) and Fooladi et al. (2014) ones [32,33].

Slight growth in the chocolate mousse samples inoculated only with free or encapsulated lactobacilli cells and the control of *E. coli* ATCC 25922 until the 7th day from the beginning of the storage under refrigerated conditions (4 ± 2 °C) was observed (Figure 2). In the controls with only free or encapsulated lactobacilli, the maximum growth rates were comparable, 0.0033 h^−1^ and 0.0038 h^−1^, respectively, while for the control of *E. coli* ATCC 25922, the maximum specific growth rate was 0.0034 h^−1^. The maximum concentration of active cells of the lactobacilli controls achieved on the 7th day was comparable and was about 10^10^ cfu/g, while that of the pathogen control was about 10^9^ cfu/g. A reduction in lactobacilli and *E. coli* ATCC 25922 cells in the controls after the 7th day was observed, and at the end of the storage period, the concentration of active lactobacilli cells in the chocolate mousse samples (inoculated with only free or encapsulated lactobacilli cells) and the *E. coli* ATCC 25922 control was the same and was about 10^7^ cfu/g. Once again, the concentration of active pathogen cells remained high and exceeded the minimum infective concentration. The death rate constants for the lactobacilli controls (inoculated with only free or encapsulated lactobacilli cells) were 0.039 h^−1^ and 0.046 h^−1^, respectively, and that of the pathogen was 0.038 h^−1^. In the mixed populations of free or encapsulated lactobacilli and *E. coli* ATCC 25922 in chocolate mousse (food matrix), there was also an increase in the active lactobacilli cells up to day 7 with the maximum specific growth rates being comparable to those of the respective controls—0.0030 h^−1^ for the lactobacilli number in the samples inoculated with free lactobacilli cells and *E. coli* ATCC 25922, and 0.0037 h^−1^ for the lactobacilli number in the variant inoculated with encapsulated lactobacilli cells and *E. coli* ATCC 25922. The achieved concentration of active lactobacilli in the mixed population was about 10^9^ cfu/g. A reduction in the lactobacilli number in the mixed population after the 7th day of storage was observed, and this trend was similar to the controls, and at the end of the storage period, the concentration of free lactobacilli in the mixed population reached about 10^9^ cfu/g, and that in the mousse inoculated with encapsulated lactobacilli cells was about 10^7^ cfu/g. The death rate constants were 0.021 h^−1^ and 0.035 h^−1^, respectively. A reduction in viable pathogen cells from the very beginning of the storage period with a death rate constant of 0.062 h^−1^ was observed in the mixed population of *E. coli* ATCC 25922 and free lactobacilli cells. At the end of the storage period, 1 cfu/g was determined. In the mixed population of *E. coli* ATCC 25922 and encapsulated lactobacilli cells, the concentration of active pathogen cells was maintained until the 7th day, after which their reduction with a death rate constant of 0.069 h^−1^ began, the death rate constant being comparable to that in the presence of the free *Lb. helveticus* 2/20 cells. Once again, 1 cfu/g of the pathogen was detected at the end of the storage process, which can be considered as complete elimination of the pathogen by LAB in the food matrix. In the mixed populations of *E. coli* ATCC 25922 and *Lb. helveticus* 2/20, it was observed that after 14 days of refrigerated storage, lactobacilli (inoculated as free or encapsulated cells) managed to reduce the population of active pathogen cells to values close to the minimum infectious concentration, about 10^6^ cfu/g, after which almost complete inactivation of *E. coli* ATCC 25922 by lactobacilli was observed (Figure 2).

**Figure 4:**
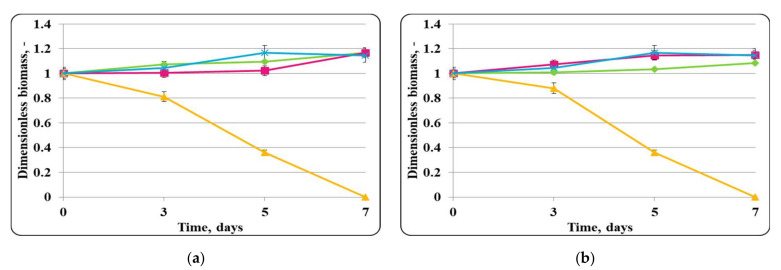
(**a**) free lactobacilli cells; (**b**) encapsulated lactobacilli cells. Changes in the dimensionless biomass of *Lb. helveticus* 2/20 and *E. coli* ATCC 25922 in chocolate mousse at 20 ± 2 °C. Legend: **Green** **◆**: *Lb. helveticus* 2/20 population following its inoculation; **Pink** **■**: *Lb. helveticus* 2/20 population following its co-inoculation with *E. coli* ATCC 25922; **Blue** **×**: *E. coli* ATCC 25922 population following its direct inoculation; **Yellow** **▲**: *E. coli* ATCC 25922 population following its co-inoculation with *Lb. helveticus* 2/20.

A continuous increase in the concentration of viable lactobacilli cells in the mixed population of free LAB and *E. coli* ATCC 25922 in the chocolate mousse stored at 20 ± 2 °C was observed a concentration of active cells of about 10^10^ cfu/g at the end of the storage period was measured (Figure 4). Maximum concentration of lactobacilli in the mixed population of encapsulated LAB and *E. coli* ATCC 25922 in chocolate mousse was reached after 5 days of storage and was about 10^9^ cfu/g, and it remained constant until the end of the storage period. The maximum specific growth rates of lactobacilli, inoculated as free or encapsulated cells, in the mixed populations with the pathogen in MRS broth were slightly reduced and remained relatively high and close to those of the controls, 0.023 h^−1^ and 0.021 h^−1^, respectively. A reduction in active pathogen cells from the beginning of the storage period, with relatively high death rates of 0.216 h^−1^ and 0.234 h^−1^, respectively, in the mixed population of *E. coli* ATCC 25922 and *Lb. helveticus* 2/20 (inoculated as free or encapsulated cells) in chocolate mousse stored at 20 ± 2 °C, was observed (Figure 4).

**Figure 5:**
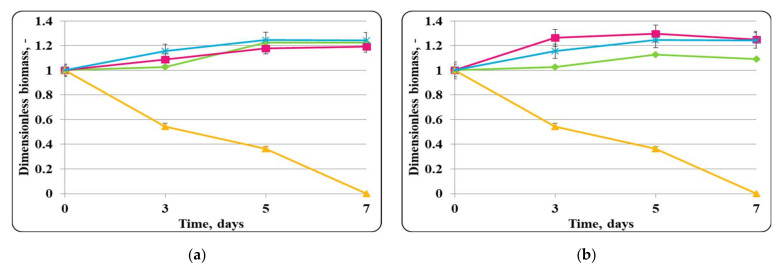
(**a**) free lactobacilli cells; (**b**) encapsulated lactobacilli cells. Changes in the population of viable cells of *Lb. helveticus* 2/20 and *E. coli* ATCC 25922 in MRS broth at 20 ± 2 °C. Legend: **Green** **◆**: *Lb. helveticus* 2/20 population following its inoculation; **Pink** **■**: *Lb. helveticus* 2/20 population following its co-inoculation with *E. coli* ATCC 25922; **Blue** **×**: *E. coli* ATCC 25922 population following its direct inoculation; **Yellow** **▲**: *E. coli* ATCC 25922 population following its co-inoculation with *Lb. helveticus* 2/20.

Unlike at 4 °C, a storage temperature of 20 ± 2 °C allowed the growth of microorganisms in MRS broth (Figure 5). In the lactobacilli controls (inoculated only with free or encapsulated *Lb. helveticus* 2/20 cells), there was a continuous increase in the active cells population in the food matrix with maximum specific growth rates of 0.026 h^−1^ and 0.029 h^−1^, respectively. Populations of active lactobacilli cells were about 10^9^–10^10^ cfu/g at the end of the storage period in both chocolate mousse controls inoculated with lactobacilli. An increase in the concentration of viable pathogen cells up to the 5th day from the beginning of the storage with a maximum growth rate of 0.051 h^−1^ was observed following chocolate mousse contamination with *E. coli* ATCC 25922 alone. The concentration of active cells of the pathogen reached about 10^12^ cfu/g, after which it remained constant until the end of the storage period. Comparatively, the concentration of active lactobacilli cells in both lactobacilli MRS broth controls increased up to the 5th day of storage, with a maximum growth rate of 0.039 h^−1^ and 0.026 h^−1^, respectively (Figure 5). These values were close to those of the respective chocolate mousse samples stored at 20 ± 2 °C, which showed that the mousse was a favorable growth environment and ensured the preservation of a high titer of beneficial probiotic microflora. An increase in the concentration of active pathogen cells up to the 5th day of storage at room temperature in the *E. coli* ATCC 25922 control inoculated in MRS broth was also observed. It reached about 10^10^ cfu/mL, after which this value remained constant until the end of the storage period. The maximum growth rate of the pathogen in MRS broth at 20 ± 2 °C was 0.053 h^−1^, which was close to that obtained in the chocolate mousse stored at 20 ± 2 °C (0.051 h^−1^) (Figure 4). This confirmed the conclusion that chocolate mousse food matrix was a suitable environment for pathogen growth, which would lead to their rapid growth above minimum infectious concentration of *E. coli* ATCC 25922, when stored at room temperature.

A similar trend was observed in the mixed populations of *Lb. helveticus* 2/20 (inoculated as free or encapsulated cells) and *E. coli* ATCC 25922 in MRS broth at 20 ± 2 °C: there was a continuous increase in the concentration of active lactobacilli cells, and the maximum specific growth rates were close to those of the respective lactobacilli controls, 0.033 h^−1^ and 0.023 h^−1^, respectively (Figure 5). A reduction in the viable pathogen cells from the beginning of the storage period, with a relatively high death rate constant of 0.144 h^−1^ in the mixed population of *E. coli* ATCC 25922 and *Lb. helveticus* 2/20 (inoculated as free or encapsulated cells) in MRS broth at storage temperature of 20 ± 2 °C, was observed (Figure 5).

The possibility of predicting the final concentrations of active cells of LAB or pathogens in chocolate mousse at different storage temperatures in the range from 4 ± 2 °C to 20 ± 2 °C is quite an interesting opportunity. Indeed, predictive microbiology mathematical models allow us to predict the evolution of microorganisms in foods and are increasingly used to ensure the microbiological safety of foods in complementarity with existing quality assurance systems [34]. For this purpose, the activation energy of growth of the lactobacilli in the mixed populations was calculated (according to equations 3 and 4) from the data on the maximum growth rates of lactobacilli, inoculated as free or encapsulated cells, and *E. coli* ATCC 25922 in the chocolate mousse samples stored at both storage temperatures (Table 3).

The activation energies of lactobacilli growth in the mixed populations with *E. coli* ATCC 25922 were comparable to those of the lactobacilli controls, which showed that the presence of the pathogen and its metabolites did not lead to *Lb. helveticus* 2/20 using additional energy for its growth and development, as well as for the processes related to its competition with the pathogen, i.e., competition for substrate consumption, synthesis of substances with antimicrobial action and its adaptation to the metabolites secreted by the pathogen (Table 3). This characterizes *Lb. helveticus* 2/20 as a microorganism with strong antimicrobial properties against the tested pathogenic strain *E. coli* ATCC 25922. When the activation energy and the pre-exponential multiplier are known, the maximum specific growth rate of lactobacilli alone (in the chocolate mousse controls inoculated with free or encapsulated *Lb. helveticus* 2/20 cells), of *E. coli* ATCC 25922 in the pathogen control or of both microorganisms in the mixed populations in chocolate mousse at different temperatures in the range from 4 ± 2 °C to 20 ± 2 °C, can theoretically be calculated according to the following equation:

(6) $$\ln N\;=\;\ln N_{0}+\;{µ}_{max}\tau$$

where *N*_0_ is the initial biomass concentration, cfu/g or mL.

**Figure 6:**
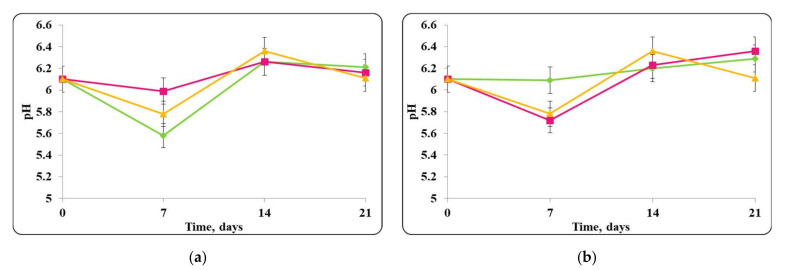
(**a**) free lactobacilli cells; (**b**) encapsulated lactobacilli cells. Changes in the pH of chocolate mousse inoculated with *Lb. helveticus* 2/20 and *E. coli* ATCC 25922 during storage at 4 ± 2 °C. Legend: **Green** **◆**: pH of chocolate mousse inoculated with *Lb. helveticus* 2/20; **Pink** **■**: pH of chocolate mousse co-inoculated with *Lb. helveticus* 2/20 and *E. coli* ATCC 25922; **Yellow** **▲**: pH of chocolate mousse inoculated with *E. coli* ATCC 25922.

**Figure 7:**
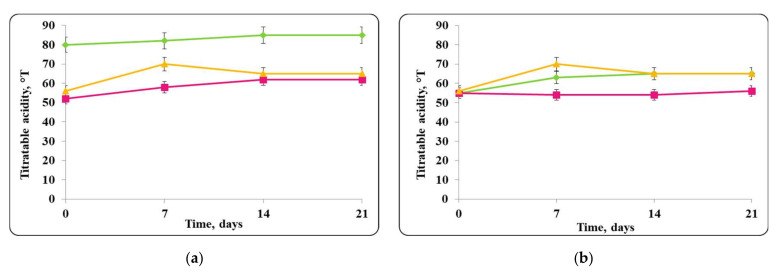
(**a**) free lactobacilli cells; (**b**) encapsulated lactobacilli cells. Changes in the total titratable acidity (TA) of MRS broth inoculated with *Lb. helveticus* 2/20 and *E. coli* ATCC 25922 during storage at 4 ± 2 °C. Legend: **Green** **◆**: TA of MRS broth inoculated with *Lb. helveticus* 2/20; **Pink** **■**: TA of MRS broth co-inoculated with *Lb. helveticus* 2/20 and *E. coli* ATCC 25922; **Yellow** **▲**: TA of MRS broth inoculated with *E. coli* ATCC 25922.

**Figure 8:**
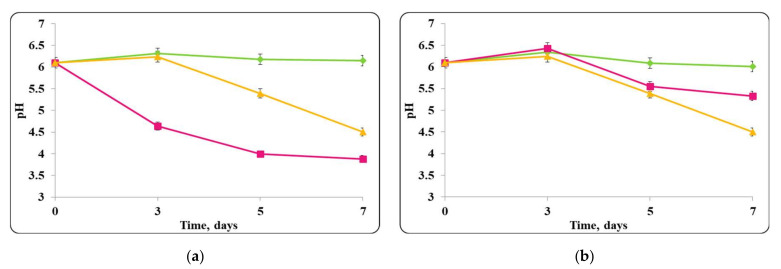
(**a**) free lactobacilli cells; (**b**) encapsulated lactobacilli cells. Changes in the pH of chocolate mousse inoculated with *Lb. helveticus* 2/20 and *E. coli* ATCC 25922 during storage at 20 ± 2 °C. Legend: **Green** ◆: pH of chocolate mousse inoculated with *Lb. helveticus* 2/20; **Pink** **■**: pH of chocolate mousse co-inoculated with *Lb. helveticus* 2/20 and *E. coli* ATCC 25922; **Yellow** **▲**: pH of chocolate mousse inoculated with *E. coli* ATCC 25922.

**Figure 9:**
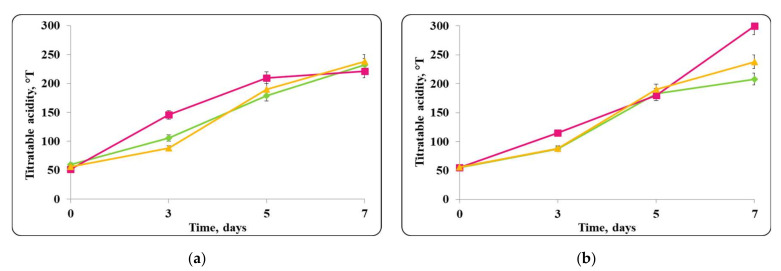
(**a**) free lactobacilli cells; (**b**) encapsulated lactobacilli cells. Changes in the total titratable acidity (TA) of MRS broth inoculated with *Lb. helveticus* 2/20 and *E. coli* ATCC 25922 during storage at 20 ± 2 °C. Legend: **Green** **◆**: TA of MRS broth inoculated with *Lb. helveticus* 2/20; **Pink** **■**: TA of MRS broth co-inoculated with *Lb. helveticus* 2/20 and *E. coli* ATCC 25922; **Yellow** **▲**: TA of MRS broth inoculated with *E. coli* ATCC 25922.

The pH of the chocolate mousse samples, stored under refrigeration conditions (4 ± 2 °C) and at room temperature (20 ± 2 °C) was also monitored (Figure 6, Figure 7, Figure 8 and Figure 9).

There was a slight change in the pH of the chocolate mousse samples during the storage period (Figure 6 and Figure 9). The change was less significant until the 7th day in the chocolate mousse samples with free *Lb. helveticus* 2/20 cells stored at 4 ± 2 °C (Figure 6), while in the samples stored at room temperature, the changes in the pH during the storage period were very significant, the reduction was 2 pH units (Figure 8). LAB such as *Lb. helveticus* are known to consume glucose and produce lactic acid when growing in glucose-containing media such as MRS broth [14], while *E. coli* has been reported to produce acetic acid in such media [15]. This would explain the pH reduction during the first 7 days of storage of the chocolate mousse with encapsulated lactobacilli cells at 4 ± 2 °C (Figure 6) and of the chocolate mousse with free or encapsulated lactobacilli cells at 20 ± 2 °C (Figure 8). The titratable acidity of the complex nutrient medium varied in the range of 50 °T to 90 °T for all MRS broth samples stored under refrigeration conditions (Figure 7). The intensive growth of the free or encapsulated cells of *Lb. helveticus* 2/20 inhibited the pathogen growth from the very beginning of the co-cultivation, reducing the concentration of viable pathogenic cells below the threshold of detection of the method for enumeration (Figure 5).

The data from the experimental studies unequivocally show that the chocolate mousse food matrix, inoculated with free or encapsulated *Lb. helveticus* 2/20 cells, preserved both the viability of lactobacilli and the pathogenic microorganisms (Figure 4). In the food matrix stored at 20 ± 2 °C, high concentrations of living cells of both the lactobacilli and the pathogen remained. The titratable acidity of MRS broth varied from 50 °T to 300 °T. Thus, the conditions for the growth of pathogenic bacteria were detrimental and likely contributed to the observed reduction in the viable pathogen counts (Figure 9).

B. Biopreservation of chocolate mousse variants with *Lb. helveticus* 2/20. Monitoring of lactobacilli and *S. aureus* ATCC 25923 populations, pH, and titratable acidity of chocolate mousse and MRS broth during storage at 4 ± 2 °C and 20 ± 2 °C.

**Figure 10:**
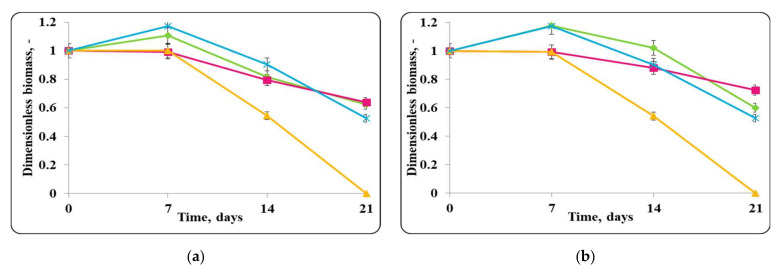
(**a**) free lactobacilli cells; (**b**) encapsulated lactobacilli cells. Changes in the dimensionless biomass of *Lb. helveticus* 2/20 and *S. aureus* ATTC 25923 in chocolate mousse at 4 ± 2 °C. Legend: **Green** **◆**: *Lb. helveticus* 2/20 population following its inoculation; **Pink** **■**: *Lb. helveticus* 2/20 population following its co-inoculation with *S. aureus* ATCC 25923; **Blue**: × *S. aureus* ATCC 25923 population following its direct inoculation; **Yellow** **▲**: *S. aureus* ATCC 25923 population following its co-inoculation with *Lb. helveticus* 2/20.

**Figure 11:**
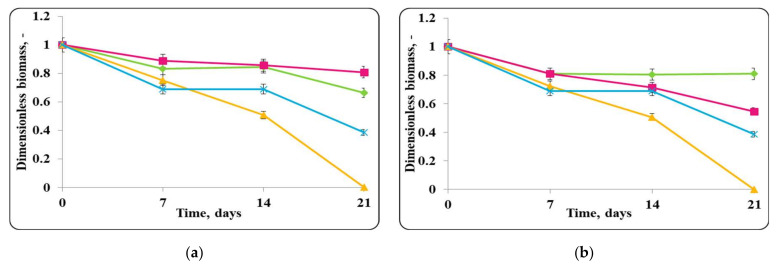
(**a**) free lactobacilli cells; (**b**) encapsulated lactobacilli cells. Changes in the dimensionless biomass of *Lb. helveticus* 2/20 and *S. aureus* ATCC 25923 in MRS broth at 4 ± 2 °C. Legend: **Green** **◆**: *Lb. helveticus* 2/20 population following its inoculation; **Pink** **■**: *Lb. helveticus* 2/20 population following its co-inoculation with *S. aureus* ATCC 25923; **Blue**: × *S. aureus* ATCC 25,923 population following its direct inoculation; **Yellow** **▲**: *S. aureus* ATCC 25923 population following its co-inoculation with *Lb. helveticus* 2/20.

Similar studies were performed with the Gram-positive pathogen strain, *S. aureus* ATCC 25923 (Figure 10 and Figure 11).

MRS broth at 4 ± 2 °C did not support the growth of *S. aureus* ATCC 25923 or *Lb. helveticus* 2/20 (Figure 11). A gradual reduction in the concentration of viable *S. aureus* ATCC 25923 cells with a death rate constant of 0.029 h^−1^ from the beginning of the storage period in MRS broth at 4 ± 2 °C was observed; at the end of the storage period, the number of viable pathogen cells was about 10^4^ cfu/mL. Complete reduction in the active cells of *S. aureus* ATCC 25923 with death rate constants of 0.057 h^−1^ and 0.055 h^−1^, respectively, in its mixtures with free or encapsulated *Lb. helveticus* 2/20 cells was observed. Reduction in *Lb. helveticus* 2/20 counts in the mixed populations of the strain (inoculated as free or encapsulated cells) and *S. aureus* ATCC 25923 in MRS broth at 4 ± 2 °C was established. The lactobacilli death rate constants were 0.01 h^−1^ and 0.02 h^−1^, respectively, their populations being about 10^6^–10^8^ cfu/mL at the end of the storage period (Figure 11).

The chocolate mousse food matrix maintained the growth of *S. aureus* ATCC 25923 and *Lb. helveticus* 2/20 to some extent (Figure 10). In the control sample inoculated with *S. aureus* ATCC 25923 alone, an increase in the number of active cells to about 10^10^ cfu/g by day 7 of the storage period, at a maximum growth rate of 0.0008 h^−1^ was observed. Then, a reduction in the pathogen active cells followed with a death rate constant of 0.049 h^−1^ and at the end of the storage period the concentration of pathogen living cells reached 10^5^ cfu/g. The growth of the pathogen in the food matrix at storage temperature of 4 ± 2 °C during storage could have been associated, on one hand, with the synthesis of staphylococcal enterotoxins and on the other hand there was no complete reduction in *S. aureus* ATCC 25923 cells, which were preserved above the level of minimum infectious concentration by the end of the storage period. A constant concentration of active cells of *S. aureus* ATCC 25923 until the 7th day of storage in the mixed populations of the pathogen and *Lb. helveticus* 2/20, inoculated as free or encapsulated cells, in the food matrix at 4 ± 2 °C was observed. Then, a reduction in the pathogen cells population with the same death rate constants (0.052 h^−1^) for its both mixtures with lactobacilli (inoculated as free or encapsulated cells) began, and at the end of the storage period, no more viable cells of the pathogen were detected (Figure 10).

There was a slight increase in and retention of a relatively constant population of active lactobacilli cells in the chocolate mousses samples inoculated with free or encapsulated cells until day 7, which was characterized by maximum specific growth rates (0.0004 h^−1^) for the samples inoculated with free or encapsulated *Lb. helveticus* 2/20 cells, in the mixed population of *Lb. helveticus* 2/20 and *S. aureus* ATCC 25923 in chocolate mousse (Figure 10). This maximum specific growth rate constant characterized the equilibrium state between the newly formed cells and the dead cells in the population. The concentration of active lactobacilli cells in the mixed population was 10^10^ cfu/g. A reduction in the lactobacilli number in the mixed population, with death rate constants of 0.028 h^−1^ and 0.021 h^−1^, respectively, for the chocolate mousse samples inoculated with free or encapsulated lactobacilli in their mixtures with *S. aureus* ATCC 25923 after the 7th day of storage, was observed. At the end of the storage period, the lactobacilli concentration in the mixed population was about 10^7^ cfu/g for the samples inoculated with free lactobacilli cells and about 10^8^ cfu/g for the samples inoculated with encapsulated lactobacilli cells (Figure 10).

**Figure 12:**
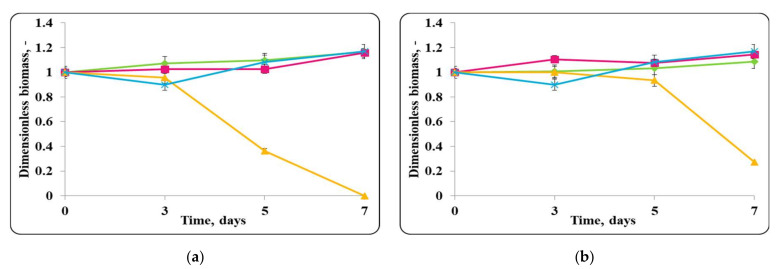
(**a**) free lactobacilli cells; (**b**) encapsulated lactobacilli cells. Changes in the dimensionless biomass of *Lb. helveticus* 2/20 and *S. aureus* ATCC 25923 in chocolate mousse at 20 ± 2 °C. Legend: **Green** **◆**: *Lb. helveticus* 2/20 population following its inoculation; **Pink** **■**: *Lb. helveticus* 2/20 population following its co-inoculation with *S. aureus* ATCC 25923; **Blue** **×**: *S. aureus* ATCC 25923 population following its direct inoculation; **Yellow** **▲**: *S. aureus* ATCC 25923 population following its co-inoculation with *Lb. helveticus* 2/20.

In the chocolate mousse pathogen control stored at 20 ± 2 °C, there was an increase in the concentration of active cells with a maximum specific growth rate of 0.023 h^−1^, and at the end of the storage period, a maximum concentration of active pathogen cells of 10^10^ cfu/g was determined (Figure 12). A steady decrease in the concentration of active pathogen cells with a death rate of 0.252 h^−1^ was observed, and no viable *S. aureus* cells were found at the end of the storage period in the mixed population of *S. aureus* ATCC 25923 and free *Lb. helveticus* 2/20 cells. While pathogen population remained constant up to the 3rd day of storage, a reduction in its population with a death rate of 0.248 h^−1^ was then observed in the mixed population of the test microorganism and encapsulated *Lb. helveticus* 2/20 cells in chocolate mousse at 20 ± 2 °C. The pathogen cell concentration at the end of the storage period was about 10^3^ cfu/g, which was two logarithmic units lower than the minimum infective concentration. The slower reduction and the higher residual population of active cells of *S. aureus* ATCC 25923 in its mixed population with encapsulated lactobacilli cells was likely due to the slower diffusion of antimicrobials through the immobilization matrix and probably the higher resistance of *S. aureus* ATCC 25923 to substances with antimicrobial activity in comparison with *E. coli* ATCC 25922.

Free and encapsulated LAB in a mixed population with *S. aureus* ATCC 25923 retained their ability to grow in chocolate mousse at a storage temperature of 20 ± 2 °C with relatively high growth rates of 0.021 h^−1^ for free cells and 0.023 h^−1^ for encapsulated cells (Figure 12). At the end of the storage period, the population of active LAB cells in the mixed populations with *S. aureus* ATCC 25923 reached values of up to about 10^10^ cfu/g. These high values of the final population of active lactobacilli cells indicated that they were not affected by the presence of *S. aureus* ATCC 25923 (Figure 12).

**Figure 13:**
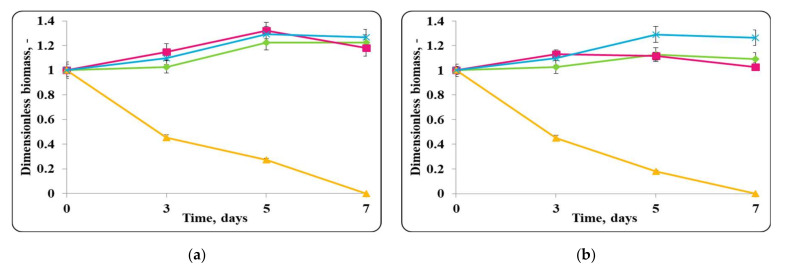
(**a**) free lactobacilli cells; (**b**) encapsulated lactobacilli cells. Changes in the dimensionless biomass of *Lb. helveticus* 2/20 and *S. aureus* ATCC 25923 in MRS broth at 20 ± 2 °C. Legend: **Green** **◆**: *Lb. helveticus* 2/20 population following its inoculation; **Pink** **■**: *Lb. helveticus* 2/20 population following its co-inoculation with *S. aureus* ATCC 25923; **Blue** **×**: *S. aureus* ATCC 25923 population following its direct inoculation; **Yellow** **▲**: *S. aureus* ATCC 25923 population following its co-inoculation with *Lb. helveticus* 2/20.

MRS broth supported the growth of the studied microorganisms at 20 ± 2 °C, unlike at 4 ± 2 °C (Figure 11 and Figure 13, respectively). In MRS broth at 20 ± 2 °C, there was an increase in the concentration of active *S. aureus* cells with a relatively high maximum specific growth rate of 0.060 h^−1^ until the 5th day of the storage period, and the population of active cells reached 10^11^ cfu/mL, and it remained relatively constant until the end of storage (Figure 13). In the mixed population of *S. aureus* ATCC 25923 and free or encapsulated *Lb. helveticus* 2/20 cells in MRS broth, there was a continuous decrease in the population of active pathogen cells from the very beginning of the storage with death rate constant values of 0.149 h^−1^ and 0.154 h^−1^, respectively, and no viable pathogen cells were detected at the end of the process (Figure 13). The complete reduction in the active *S. aureus* cells in the mixed population with encapsulated *Lb. helveticus* 2/20 cells in contrast to that in the respective chocolate mousse was likely due to the lower viscosity of MRS broth, which is associated with better molecular and convective diffusion of the substances with antimicrobial action synthesized by *Lb. helveticus* 2/20 in the medium. In addition, MRS broth has a lower buffering capacity than chocolate mousse, and accordingly, *S. aureus* cells were exposed to a lower pH and thereby a higher antimicrobial effect of lactic acid produced (Figure 11 and Figure 13).

In the mixed population of free *Lb. helveticus* 2/20 cells and *S. aureus* ATCC 25923 in MRS broth at 20 ± 2 °C, an increase in the population of active lactobacilli cells up to day 5 with a maximum specific growth rate of 0.035 h^−1^ was observed. It reached 10^10^ cfu/mL and remained relatively constant until the end of the storage period (Figure 13). In the mixed population of encapsulated *Lb. helveticus* 2/20 cells and *S. aureus* ATCC 25923 in MRS broth stored at 20 ± 2 °C, an increase in the number of active lactobacilli cells up to day 3 with a maximum specific growth rate of 0.026 h^−1^ was observed, and the final population was 10^12^ cfu/mL. A reduction in active cells population was then observed, but it remained higher than 10^11^ cfu/mL until the end of the storage period (Figure 13).

The activation energy of growth and the pre-exponential multiplier in the Arrhenius equation for *Lb. helveticus* 2/20 and *S. aureus* ATCC 25923 when cultured alone or in a mixed population in chocolate mousse were calculated (Table 4).

The activation energies of growth and the pre-exponential multipliers for *Lb. helveticus* 2/20 inoculated as free or encapsulated cells in its mixed population with *S. aureus* ATTC 25923 were significantly higher than those of the respective lactobacilli controls (Table 4). This indicated that the presence of *Lb. helveticus* 2/20 and its metabolites had an effect on the lactobacilli themselves, and it was associated with *Lb. helveticus* 2/20 conducting additional activity and spending more energy in order to grow and develop, and also to suppress the pathogen growth and to adapt to the metabolites secreted by *S. aureus* ATCC 25923. Higher activation energy of growth for lactobacilli in the mixed population of encapsulated *Lb. helveticus* 2/20 cells and *S. aureus* ATCC 25923 (182.7 kJ/mol) than that in the mixed population of free *Lb. helveticus* 2/20 cells and *S. aureus* ATCC 25923 (171.7 kJ/mol) was observed (Table 3). This was probably due to secreted metabolites by *S. aureus* ATCC 25923 that pass through the immobilization matrix and concentrate in the microenvironment around the LAB, which is necessary in order to activate additional enzyme systems to eliminate their action, which in its turn is associated with more energy expenditure.

The activation energies of death of *S. aureus* ATCC 25923 and *E. coli* ATCC 25922 in the chocolate mousse food matrix as well as the pre-exponential multiplier in the Arrhenius equation were also calculated (Table 5).

*S. aureus* ATCC 25923 was characterized by a slightly higher activation energy of death compared to *E. coli* ATCC 25922, which is consistent with the observation that *S. aureus* ATCC 25923 is more resistant to the presence of lactobacilli and their antimicrobial metabolites than *E. coli* ATCC 25922. This once again confirmed that *Lb. helveticus* 2/20 from the mixed population of *Lb. helveticus* 2/20 and *S. aureus* ATCC 25923 will need to spend more energy to completely reduce the amount of *S. aureus* ATCC 25923 cells, compared to the energy required for the complete reduction in *E. coli* ATCC 25922.

With these parameters, it is possible to determine the death rate and the time for complete reduction in pathogen cells at different storage temperatures ranging from 4 ± 2 °C to 20 ± 2 °C.

**Figure 14:**
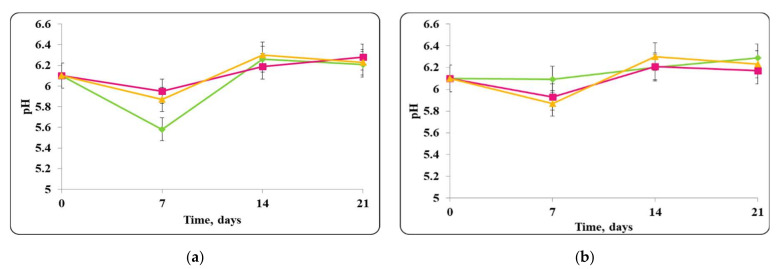
(**a**) free lactobacilli cells; (**b**) encapsulated lactobacilli cells. Changes in the pH of the chocolate mousse inoculated with *Lb. helveticus* 2/20 and *S. aureus* ATTC 25923 during storage at 4 ± 2 °C. Legend: **Green** **◆**: pH of chocolate mousse inoculated with *Lb. helveticus* 2/20; **Pink** **■**: pH of chocolate mousse co-inoculated with *Lb. helveticus* 2/20 and *S. aureus* ATCC 25923; **Yellow** **▲**: pH of chocolate mousse inoculated with *S. aureus* ATCC 25923.

**Figure 15:**
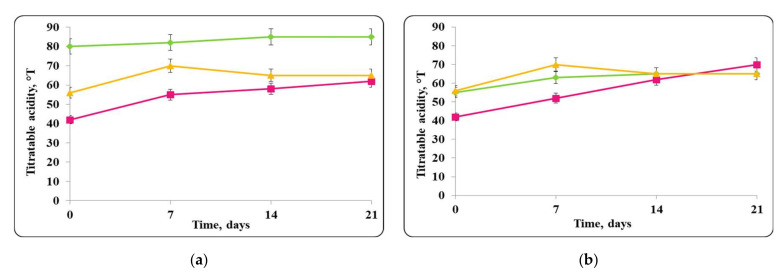
(**a**) free lactobacilli cells; (**b**) encapsulated lactobacilli cells. Changes in the total titratable acidity of MRS broth inoculated with *Lb. helveticus* 2/20 and *S. aureus* ATTC 25923 during storage at 4 ± 2 °C. Legend: **Green** **◆**: TA of MRS broth inoculated with *Lb. helveticus* 2/20; **Pink** **■**: TA of MRS broth co-inoculated with *Lb. helveticus* 2/20 and *S. aureus* ATCC 25923; **Yellow** **▲**: TA of MRS broth inoculated with *S. aureus* ATCC 25923.

**Figure 16:**
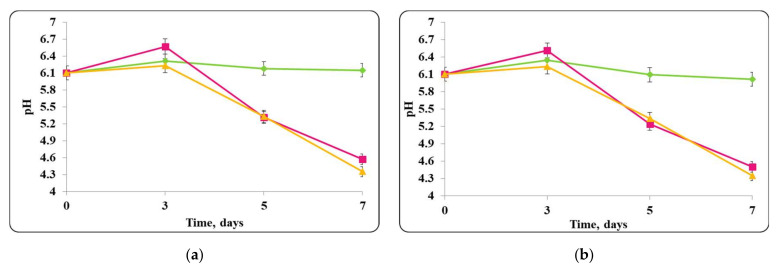
(**a**) free lactobacilli cells; (**b**) encapsulated lactobacilli cells. Changes in the pH of the chocolate mousse inoculated with *Lb. helveticus* 2/20 and *S. aureus* ATTC 25923 during storage at 20 ± 2 °C. Legend: **Green** **◆**: pH of chocolate mousse inoculated with *Lb. helveticus* 2/20; **Pink** **■**: pH of chocolate mousse co-inoculated with *Lb. helveticus* 2/20 and *S. aureus* ATCC 25923; **Yellow** **▲**: pH of chocolate mousse inoculated with *S. aureus* ATCC 25923.

**Figure 17:**
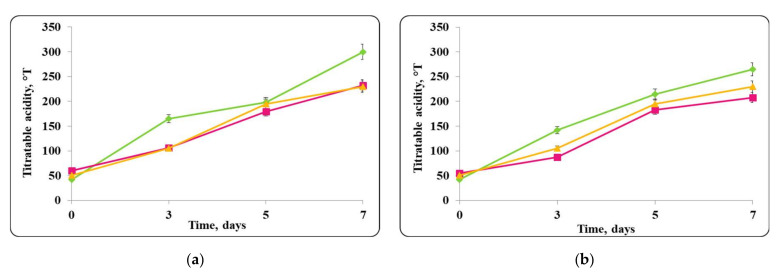
(**a**) free lactobacilli cells; (**b**) encapsulated lactobacilli cells. Changes in the total titratable acidity (TA) of MRS broth inoculated with *Lb. helveticus* 2/20 and *S. aureus* ATTC 25923 during storage at 20 ± 2 °C. Legend: **Green** **◆**: TA of MRS broth inoculated with *Lb. helveticus* 2/20; **Pink** **■**: TA of MRS broth mousse co-inoculated with *Lb. helveticus* 2/20 and *S. aureus* ATCC 25923; **Yellow** **▲**: TA of MRS broth inoculated with *S. aureus* ATCC 25923.

The changes in pH of the finished mousses during storage period at 20 ± 2 °C were monitored as well (Figure 14, Figure 15, Figure 16 and Figure 17).

A pH change in the first days following the preparation of the chocolate mousse samples preserved with free or encapsulated *Lb. helveticus* 2/20 cells, contaminated with *S. aureus* ATCC 25923 and stored at 4 ± 2 °C, was observed (Figure 14). During further storage, a relatively constant pH value was maintained until the end of the storage period. For the chocolate mousse samples containing only free or encapsulated *Lb. helveticus* 2/20 cells (controls), the pH values were 5.99 and 6.3 and remained within these limits throughout the storage period. A slight increase in the pH was observed in the mixtures of free or encapsulated cells of *Lb. helveticus* 2/20 and *S. aureus* ATCC 25923. The pH of the chocolate mousse samples with free *Lb. helveticus* 2/20 cells and *S. aureus* ATCC 25923 increased from 6.0 to 6.3 during the storage period, and for the chocolate mousse samples with encapsulated *Lb. helveticus* 2/20 cells and *S. aureus* ATCC 25923, it increased from 5.9 to 6.2 (Figure 14).

When the pathogen was co-cultivated with *Lb. helveticus* 2/20, the change in the acidity of the medium was from 40 °T to 90 °T. This made the conditions for the growth of *S. aureus* ATCC 25923 more detrimental. Together with the substances with antimicrobial action secreted in the medium by *Lb. helveticus* 2/20 cells, this likely contributed to complete reduction in the living pathogen cells observed after 7 days (Figure 15).

A decrease in chocolate mousse samples pH only with free or encapsulated *Lb. helveticus* 2/20 cells, stored at a temperature of 20 ± 2 °C during the storage period, was observed (Figure 16). The pH decreased from pH = 6 to pH = 4.2 in both samples. A similar pH decrease was observed in the chocolate mousse variants with a mixed population of free or encapsulated *Lb. helveticus* 2/20 cells and *E. coli* ATCC 25922 or *S. aureus* ATCC 25923 (Figure 16).

The curves reflecting the changes in the acidity of the complex nutrient medium (MRS broth) for the co-cultures of *Lb. helveticus* 2/20 and *S. aureus* ATCC 25923 at 20 ± 2 °C looked different (Figure 17). The pathogenic staphylococci grew poorly in MRS broth. The slight change in the pH of the medium also supported the high concentration of viable *S. aureus* ATCC 25923 cells. In the co-cultivation of the pathogen with *Lb. helveticus* 2/20, the change in the acidity of the medium from 50 °T to 300 °T was significant. This made the conditions for the growth of *S. aureus* ATCC 25923 more detrimental. Together with the substances with antimicrobial action secreted in the medium, this is likely the reason for the radical reduction in the living pathogen cells population (Figure 17).

Sensorial analysis of both lactobacilli control samples (with free or encapsulated *Lb. helveticus* 2/20 cells) immediately after preparation has been performed, and it showed that there was no influence of the added probiotic strain on the taste, aroma and overall acceptance of the chocolate mousse variants by the consumers (unpublished data).

## 4. Conclusions

Biopreservation is a natural way to protect foods from microbial spoilage and harmful microbial contaminations. The study demonstrated that the use of free or encapsulated *Lb. helveticus* 2/20 cells for biopreservation in chocolate mouse is a promising hurdle to reduce pathogen survival. In addition, the results of the in vivo and in vitro assays show that the inhibition in MRS broth was similar to the one observed in the chocolate mousse stored at 20 ± 2 °C. Nevertheless, the pathogen inhibition effect observed at 4 ± 2 °C was different between in vitro and in situ assays (i.e., in MRS broth and in chocolate mousse, respectively). In the food matrix, the pathogen inhibition effect was smaller than in the nutrient medium. This is a confirmation of the fact that the use of in situ evaluation is necessary before confirming the biopreservation potential of any bioprotector. The application of the mathematical models used make it possible to determine the death rate and the time for complete reduction in pathogen cells at different storage temperatures ranging from 4 ± 2 °C to 20 ± 2 °C. Future research will focus on revealing the mechanisms of inhibition of *E. coli* ATCC 25922 and *S. aureus* ATCC 25923 growth by *Lb. helveticus* 2/20 strain (e.g., antibacterial metabolites production (lactic acid, bacteriocins and others), competition for nutrients or space) and examination of the applicability of *Lb. helveticus* 2/20 for inclusion in other biopreservation strategies for different food products.

## Figures and Tables

**Table 1:** Samples of chocolate mousse with lactobacilli and pathogenic bacteria.

№	Chocolate Mousse Composition
1	Chocolate mousse control
2	Chocolate mousse contaminated with *S. aureus* ATCC 25923 cells alone
3	Chocolate mousse contaminated with *E. coli* ATCC 25922 cells alone
4	Chocolate mousse inoculated with free *Lb. helveticus* 2/20 cells
5	Chocolate mousse inoculated with encapsulated *Lb. helveticus* 2/20 cells
6	Chocolate mousse inoculated with free *Lb. helveticus* 2/20 cells and contaminated with *S. aureus* ATCC 25923 cells
7	Chocolate mousse inoculated with encapsulated *Lb. helveticus* 2/20 cells and contaminated with *S. aureus* ATCC 25923 cells
8	Chocolate mousse inoculated with free *Lb. helveticus* 2/20 cells and contaminated with *E. coli* ATCC 25922 cells
9	Chocolate mousse inoculated with encapsulated *Lb. helveticus* 2/20 cells and contaminated with *E. coli* ATCC 25922 cells

**Table 2:** Ability of *Lactobacillus* 2/20 to utilize the 49 carbon sources included in the API 50 CHL identification system.

#	Carbon Source	*Lactobacillus* 2/20
1	Glycerol	-
2	Erythriol	-
3	D-arabinose	-
4	L-arabinose	-
5	Ribose	-
6	D-xylose	-
7	L-xylose	-
8	Adonitol	-
9	β-metil-D-xyloside	-
10	Galactose	+ (90–100%)
11	D-glucose	+ (90–100%)
12	D-fructose	+ (90–100%)
13	D-mannose	+ (90–100%)
14	L-sorbose	-
15	Rhamnose	-
16	Dulcitol	-
17	Inositol	-
18	Manitol	+ (90–100%)
19	Sorbitol	+ (90–100%)
20	α-methyl-D-mannoside	+ (90–100%)
21	α-methyl-D-glucoside	+ (90–100%)
22	*N*-acetyl-glucosamine	+ (90–100%)
23	Amigdalin	+ (90–100%)
24	Arbutin	+ (90–100%)
25	Esculin	+ (90–100%)
26	Salicin	+ (90–100%)
27	Cellobiose	+ (90–100%)
28	Maltose	+ (90–100%)
29	Lactose	+ (90–100%)
30	Melibiose	+ (90–100%)
31	Saccharose	+ (90–100%)
32	Trehalose	+ (90–100%)
33	Inulin	+ (90–100%)
34	Melezitose	+ (90–100%)
35	D-raffinose	+ (90–100%)
36	Amidon	+ (90–100%)
37	Glycogen	-
38	Xylitol	-
39	β-gentiobiose	+ (90–100%)
40	D-turanose	+ (90–100%)
41	D-lyxose	-
42	D-tagarose	-
43	D-fuccose	-
44	L-fuccose	-
45	D-arabitol	-
46	L-arabitol	-
47	Gluconate	+ (90–100%)
48	2-keto-gluconate	-
49	5-keto-gluconate	-

**Table 3:** Activation energy of growth and pre-exponential multiplier in the Arrhenius equation for *Lb. helveticus* 2/20 and *E. coli* ATCC 25922 in chocolate mousse.

Microorganisms	Ea (kJ/mol)	Z (h^−1^)
Free *Lb. helveticus* 2/20 cells	87	8.6 × 10^13^
Encapsulated *Lb. helveticus* 2/20 cells	85.9	5.9 × 10^13^
Free *Lb. helveticus* 2/20 cells in co-culture with *E. coli* ATCC 25922	85.4	3.9 × 10^13^
Encapsulated *Lb. helveticus* 2/20 cells in co-culture with *E. coli* ATCC 25922	86.1	6.4 × 10^13^
*E. coli* ATCC 25922	116.2	2.7 × 10^13^

**Table 4:** Activation energy of growth and pre-exponential multiplier in the Arrhenius equation for *Lb. helveticus* 2/20 and *S. aureus* ATCC 25923 in chocolate mousse.

Microorganisms	Ea k(J/mol)	Z (h^−1^)
Free *Lb. helveticus* 2/20 cells	87	8.6 × 10^13^
Encapsulated *Lb. helveticus* 2/20 cells	85.9	5.9 × 10^13^
Free *Lb. helveticus* 2/20 cells in co-culture with *S. aureus* ATCC 25923	171.7	8.6 × 10^28^
Encapsulated *Lb. helveticus* 2/20 cells in co-culture with *S. aureus* ATCC 25923	182.7	1.1 × 10^31^
*S. aureus* ATCC 25923	140.2	2.3 × 10^23^

**Table 5:** Activation energy of death and pre-exponential multiplier in the Arrhenius equation for *S. aureus* ATCC 25923 and *E. coli* ATCC 25922 in chocolate mousse.

Strain/Mixture	Ea (kJ/mol)	Z (h^−1^)
*E. coli* ATCC 25922 in co-culture with *Lb. helveticus* 2/20 (free cells)	50.9	3.0 × 10^8^
*E. coli* ATCC 25922 in co-culture with *Lb. helveticus* 2/20 (encapsulated cells)	50.2	2.2 × 10^8^
*S. aureus* ATCC 25923 in co-culture with *Lb. helveticus* 2/20 (free cells)	66.6	1.8 × 10^11^
*S. aureus* ATCC 25923 in co-culture with *Lb. helveticus* 2/20 (encapsulated cells)	65.9	1.4 × 10^11^

## Supplementary Materials

The following supporting information can be downloaded at: https://www.mdpi.com/article/10.3390/molecules27175631/s1, Table S1. Kinetic characteristics of the microbial population.

## Sample Availability

Samples of the compounds are not available from the authors.
