# Supplementary material for: Biopreservation of Chocolate Mousse with Lactobacillus helveticus 2/20: Microbial Challenge Test
Source: Molecules. 2022 Aug 31;27(17):5631. doi: 10.3390/molecules27175631 (PMC9457945; doi:10.3390/molecules27175631)
Supplement: Supplementary file 1 [file molecules-27-05631-s001.zip › molecules-1884600-supplementary.pdf]

Table S1. Kinetic characteristics of the microbial population.

Sample	μ_{max}, h^{-1}, Chocolate mousse		κ, h^{-1}, Chocolate mousse	
	4±2 °C	20±2 °C	4±2 °C	20±2 °C
Free *Lb. helveticus* 2/20, direct inoculation	0,0033	0,026	0,039	-
Encapsulated *Lb. helveticus* 2/20 cells, direct inoculation	0,0038	0,029	0,046	-
E. coli ATCC 25922, direct inoculation	0,0034	0,051	0,038	-
S. aureus ATTC 25923, direct inoculation	0,0008	0,023	0,049	-
Free *Lb. helveticus* 2/20 cells in co-inoculation with *E. coli* ATCC 25922	0,0030	0,023	0,021	-
Encapsulated *Lb. helveticus* 2/20 cells in co-inoculation with *E. coli* ATCC 25922	0,0037	0,021	0,035	-
E. coli ATCC 25922 in co-inoculation with free *Lb. helveticus* 2/20 cells	-	-	0,062	0,216
E. coli ATCC 25922 in co-inoculation with encapsulated *Lb. helveticus* 2/20 cells	-	-	0,069	0,234
Free *Lb. helveticus* 2/20 cells in co-inoculation with *S. aureus* ATTC 25923	0,0004	0,021	0,028	-
Encapsulated *Lb. helveticus* 2/20 cells in co-inoculation with *S. aureus* ATTC 25923	0,0004	0,023	0,021	-
S. aureus ATTC 25923 in co-inoculation with free *Lb. helveticus* 2/20 cells	-	-	0,052	0,252
S. aureus ATTC 25923 in co-inoculation with encapsulated *Lb. helveticus* 2/20 cells	-	-	0,052	0,248
Sample	μ_{max}, h^{-1}, MRS broth		κ, h^{-1}, MRS broth	
	4±2 °C	20±2 °C	4±2 °C	20±2 °C
Free *Lb. helveticus* 2/20, direct inoculation	-	0,039	0,012	-
Encapsulated *Lb. helveticus* 2/20 cells, direct inoculation	-	0,026	0,009	-
E. coli ATCC 25922, direct inoculation	-	0,053	0,025	-
S. aureus ATTC 25923, direct inoculation	-	0,060	0,029	-
Free *Lb. helveticus* 2/20 cells in co-inoculation with *E. coli* ATCC 25922	-	0,033	0,017	-
Encapsulated *Lb. helveticus* 2/20 cells in co-inoculation with *E. coli* ATCC 25922	-	0,023	0,017	-
E. coli ATCC 25922 in co-inoculation with free *Lb. helveticus* 2/20 cells	-	-	0,062	0,144
E. coli ATCC 25922 in co-inoculation with encapsulated *Lb. helveticus* 2/20 cells	-	-	0,067	0,144

Free *Lb. helveticus* 2/20 cells in co-inoculation with *S. aureus* ATTC 25923	-	0,035	0,01	-
Encapsulated *Lb. helveticus* 2/20 cells in co-inoculation with *S. aureus* ATTC 25923	-	0,026	0,02	-
S. aureus ATTC 25923 in co-inoculation with free *Lb. helveticus* 2/20 cells	-	-	0,057	0,149
S. aureus ATTC 25923 in co-inoculation with encapsulated *Lb. helveticus* 2/20 cells	-	-	0,055	0,154
